# Prediction of Stroke Outcomes in a Super-aged Society: Different Effects Among Predictive Variables

**DOI:** 10.7759/cureus.104580

**Published:** 2026-03-02

**Authors:** Yoshihiro Kanata, Yuki Uchiyama, Satoko Matsushima, Mika Kanatani, Tetsuo Koyama, Kazuhisa Domen

**Affiliations:** 1 Department of Rehabilitation Medicine, General Medicine and Community Health, Hyogo Medical University Sasayama Medical Center, Tamba-Sasayama, JPN; 2 Department of Rehabilitation Medicine, School of Medicine, Hyogo Medical University, Nishinomiya, JPN; 3 Department of Rehabilitation Medicine, Hyogo Medical University Sasayama Medical Center, Tamba-Sasayama, JPN; 4 Department of Rehabilitation Medicine, Nishinomiya Kyoritsu Neurosurgical Hospital, Nishinomiya, JPN

**Keywords:** aging society, functional independence measure, japanese geriatrics, late elderly, stroke fehabilitation, stroke outcome

## Abstract

Background and objective: Population aging has progressed rapidly worldwide, particularly in developed countries such as Japan. Age is a well-established prognostic factor after stroke; however, its influence on functional outcomes may differ in increasingly older populations. This study aims to investigate whether the impact of age on post-stroke functional independence differs between patients aged ≥75 years (late elderly) and those aged <75 years.

Methods: This retrospective cohort study included 169 post-stroke patients admitted to a convalescent rehabilitation ward between 2018 and 2023. All patients were independent before stroke onset and received intensive multidisciplinary rehabilitation. Functional outcomes were assessed using the Functional Independence Measure (FIM), with the motor score at discharge as the primary endpoint. Patients were divided into a younger group referred to as the 'early elderly' (<75 years, n = 80) and an older group known as the 'late elderly' (≥75 years, n = 89). Multivariable linear regression analyses were performed for the entire cohort and separately for each age group, adjusting for demographic, clinical, and functional variables at admission.

Results: Baseline FIM motor and cognitive scores and trunk function were significantly poorer in the late elderly group; however, no significant age-related difference in FIM gain was observed. In the overall cohort, age, time from stroke onset to transfer, FIM cognitive score at admission, and trunk function independently predicted the FIM motor score at discharge. In subgroup analyses, age was not a significant predictor in the early elderly group but was a strong independent predictor in the late elderly group, along with cognitive function, motor impairment, trunk function, and time to rehabilitation.

Conclusions:The prognostic significance of age differs by age group in post-stroke rehabilitation. Age does not independently predict functional independence in patients aged <75 years, but plays a critical role among those aged ≥75 years. These findings highlight the need for age-specific prognostic models and rehabilitation planning in aging societies, particularly among patients undergoing intensive convalescent rehabilitation.

## Introduction

Advances in public health and medical care have increased life expectancy worldwide, leading to rapid population aging. This demographic shift is particularly pronounced in developed countries. Japan is one of the most rapidly aging societies, with individuals aged ≥65 years accounting for more than 29% of the total population. In accordance with Japanese healthcare and social insurance policy, adults aged ≥65 years are classified as 'older,' those aged 65 to 74 years as 'early elderly,' and those aged ≥75 years as 'late elderly' [1]. This distinction reflects recognized differences in health status, functional reserve, and healthcare utilization between early and late elderly populations and provides a clinically meaningful framework for age-stratified analyses of functional outcomes.

Stroke is a leading cause of disability among older adults [2]. Residual hemiparesis and cognitive impairment are common after stroke and often result in substantial limitations in independence in activities of daily living (ADL) [3]. Early initiation of rehabilitation is essential for promoting functional recovery and independence [4]. Therefore, accurate prognostic prediction is critical for establishing appropriate rehabilitation strategies [5]. For example, when independent ambulation is unlikely, rehabilitation programs emphasize basic self-care activities such as eating, grooming, and dressing. In contrast, patients expected to regain ambulatory function may be assigned more advanced goals, including stair climbing. Numerous factors influence stroke outcomes, including initial stroke severity, lesion volume, and age, the latter of which has consistently been identified as a major determinant of prognosis [6].

However, the influence of age on stroke outcomes may differ from that reported in earlier studies, particularly in developed countries experiencing extreme population aging. Accordingly, this study investigated the impact of age on functional outcomes after stroke by comparing late elderly patients with their younger counterparts in a cohort from a rural region of Japan, where population aging is especially pronounced.

## Materials and methods

Study design and participants

This retrospective cohort study included post-stroke patients admitted to our convalescent rehabilitation ward between November 2018 and March 2023. During hospitalization, patients received up to 180 minutes per day of physical therapy, occupational therapy, and speech therapy, seven days per week, in accordance with the Japanese Guidelines for the Management of Stroke [7].

Eligibility criteria included a pre-stroke modified Rankin Scale (mRS) score of ≤2, indicating independence in ambulation and activities of daily living [8], and the absence of severe dementia. As in our previous studies [9-11], patients with subarachnoid hemorrhage or lesions involving the cerebellum or brainstem were excluded, as were those with medical complications requiring acute care (e.g., angina, gastrointestinal disease, or fractures). To minimize the influence of white matter lesions, only patients with a Fazekas score <2 were included [11,12]. The Fazekas scores were evaluated using MRI by an experienced physiatrist. The mRS was used solely as an eligibility criterion and is appropriately cited. No scale items, structured interview materials, or copyrighted content related to the mRS are reproduced in this manuscript.

Functional assessment

Functional status was assessed using the Functional Independence Measure (FIM) [13]. The FIM consists of 13 motor items and five cognitive items, each scored on a 7-point scale ranging from 1 (total assistance) to 7 (complete independence). Total scores range from 18 to 126, with motor subscale scores ranging from 13 to 91 and cognitive subscale scores from 5 to 35. Discharge was determined when the FIM motor score plateaued.

Functional improvement was quantified as FIM gain, defined as the change in the FIM motor score between admission and discharge, and was used as an indicator of rehabilitation effectiveness. The FIM was used solely for routine clinical scoring and outcome reporting. No copyrighted test forms, manuals, instructions, or item-level content are reproduced or described in this manuscript.

Assessment of motor and trunk function

Motor impairment was evaluated using the motor domain of the Stroke Impairment Assessment Set (SIAS), and trunk function was assessed using the SIAS trunk items [14]. Motor function for five components (upper limb, hand/fingers, hip, knee, and ankle) was scored on a 6-point scale (0-5). The sum of these components was calculated to assess the overall severity of motor impairment in the affected extremities (SIAS motor total).

Trunk function was scored from 0 to 6 based on vertical sitting balance and abdominal muscle strength (SIAS trunk), in accordance with the SIAS scoring system. The SIAS was used in accordance with standard academic practice; only summary scores were analyzed and reported, and no proprietary materials were reproduced.

Statistical analysis

Collected data included age, sex, type of stroke (ischemic (I) or hemorrhagic (H)), time from stroke onset to transfer, FIM and SIAS scores at admission and discharge, FIM gain, and total length of hospital stay (including the acute care period). The FIM motor score reflects functional performance [13], whereas SIAS scores assess neurological impairment [14]; therefore, these variables were considered complementary rather than redundant despite assessing related domains. Prior to age stratification, age was analyzed as a continuous variable in exploratory models to evaluate potential age-dependent changes in prognostic relationships.

Statistical analyses were performed in two steps. First, participants were divided into early elderly (<75 years) and late elderly (≥75 years) groups, and group differences were examined using Wilcoxon rank-sum tests or chi-squared tests, as appropriate. Continuous variables are presented as medians with interquartile ranges and were compared using the Wilcoxon rank-sum test due to non-normal distributions. Second, multiple linear regression analyses were conducted using the forced-entry method, with the FIM motor score at discharge as the dependent variable. Independent variables included age, type of stroke, time from onset to transfer, FIM motor and cognitive scores at admission, and SIAS motor total and SIAS trunk scores at admission.

Three regression models were constructed: one including all participants, one including only the early elderly group, and one including only the late elderly group. All statistical analyses were performed using the JMP software (SAS Institute Inc., Cary, NC, USA). A p-value <0.05 was considered statistically significant.

## Results

Patient selection and baseline characteristics

**Figure 1:**
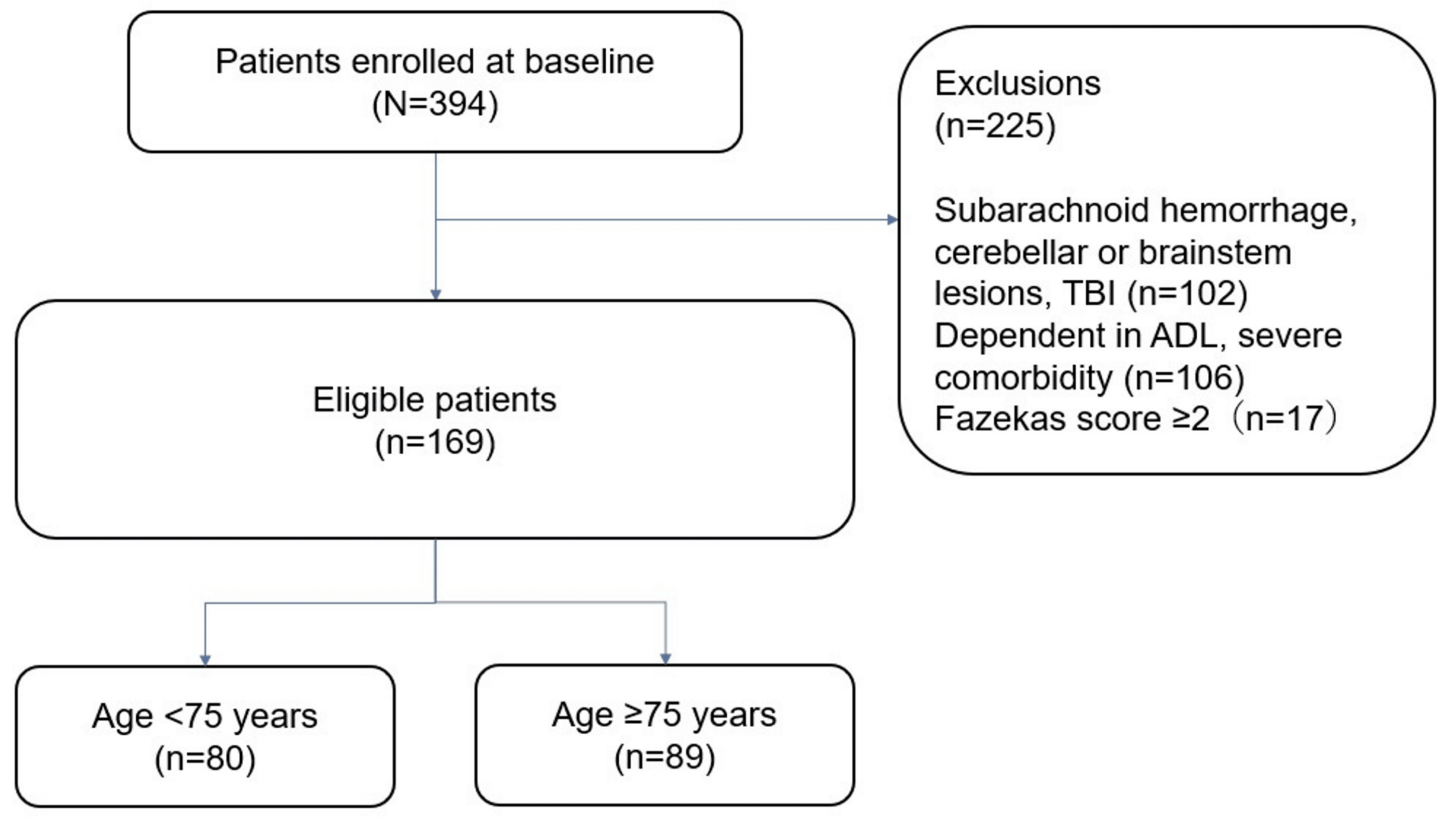
Flow diagram showing the patient screening process ADL: Activities of daily living; TBI: Traumatic brain injury

The patient selection process is summarized in Figure 1. During the study period, 394 patients were admitted to the convalescent rehabilitation ward, of whom 169 met the eligibility criteria. Based on the predefined age cutoff of 75 years, 80 patients were assigned to the early elderly group and 89 to the late elderly group. Patient characteristics are presented in Table 1.

**Table 1:** Patient demographic and clinical characteristics Data are presented as median (interquartile range) or number, as appropriate. Continuous variables were compared using the Wilcoxon rank-sum test, and categorical variables were compared using the chi-squared test. Statistically significant findings (p < 0.05) are marked with an asterisk (*). FIM: Functional Independence Measure; H: Hemorrhagic; I: Ischemic; SIAS: Stroke Impairment Assessment Set

Characteristics	Total (n=169)	Early elderly group <75 years (n=80)	Late elderly group ≥75 years (n=89)	Test statistic	p-value
Age (years)*	76 (67-83)	66.5 (59.5-71)	83 (79-89)	z = -11.21	< 0.001
Sex (M/F)*	98/71	57/23	41/48	χ² = 11.139	< 0.001
Type of stroke (H/I)	62/107	34/46	28/61	χ² = 2.212	0.137
Days from onset	27 (18-38)	27.5 (17-41)	26 (18-36.5)	z = 0.068	0.946
FIM motor at admission*	38 (19.5-58)	50.5 (27.5-67)	34 (17-49.5)	z = 3.317	< 0.001
FIM cognitive at admission*	25 (15-32)	27.5 (17.5-33)	23 (14-28)	z = 2.966	0.003
SIAS motor total at admission	15 (6.5-21)	13 (6-22.75)	16 (7-20)	z = -0.129	0.897
SIAS trunk at admission*	5 (3-6)	6 (4-6)	4 (2-6)	z = 3.563	< 0.001
Total hospital stay (days)	118 (77.5-172)	111.5 (62-170.75)	124 (85-173)	z = -1.124	0.261
FIM motor at discharge*	78 (48.5-87)	82 (72.25-88)	68 (31-82)	z = 4.102	< 0.001
FIM cognitive at discharge*	29 (22-34)	32.5 (26-35)	26 (19-33)	z = 3.948	< 0.001
SIAS motor total at discharge	19 (10.5-23)	19 (11-23)	19 (8.5-21.5)	z = 0.894	0.371
FIM gain	25 (10-39)	27.5 (12-41)	25 (5.5-36)	z = 1.036	0.300

There were no significant differences between groups in type of stroke, time from stroke onset to transfer, length of hospital stay, SIAS motor total score, or FIM gain. However, FIM motor and cognitive scores and SIAS trunk scores at admission were significantly lower in the late elderly group. A significant difference in sex distribution was observed between groups.

Predictors of functional outcome in the overall cohort

Table 2 features the multivariable linear regression analysis, including the identified age of all participants, time from stroke onset to transfer, FIM cognitive score at admission, and SIAS trunk score at admission as significant independent predictors of the FIM motor score at discharge.

**Table 2:** Results for FIM motor at discharge obtained by multivariate regression analysis Statistically significant findings (p<0.05) are marked with an asterisk (*). FIM: Functional Independence Measure; SE: Standard error; VIF: Variance inflation factor

Parameters	Estimate	SE	t	p-value	VIF
Age*	-0.354	0.100	-3.53	0.0005	1.419
Type of stroke (H/I)	3.252	2.317	1.40	0.1625	1.115
Days from onset*	-0.163	0.055	-2.95	0.0037	1.146
FIM motor at admission	0.073	0.096	0.76	0.4474	4.181
FIM cognitive at admission*	0.533	0.173	3.07	0.0025	2.214
SIAS motor total at admission	0.435	0.229	1.90	0.0593	3.111
SIAS trunk at admission*	5.490	0.996	5.51	<0.001	3.740

Age-stratified multivariable regression analyses

In the early elderly group (Table 3), time from stroke onset to transfer and SIAS trunk score at admission were significant predictors of the FIM motor score at discharge, whereas age, type of stroke, FIM motor and cognitive scores at admission, and SIAS motor total score were not. In the late elderly group (Table 4), age, time from stroke onset to transfer, FIM cognitive score at admission, SIAS motor total score, and SIAS trunk score at admission were independently associated with the FIM motor score at discharge.

**Table 3:** Results for FIM motor at discharge obtained by multivariate regression analysis in the early elderly group (aged <75 years) Statistically significant findings (p<0.05) are marked with an asterisk (*). FIM: Functional Independence Measure; SE: Standard error; SIAS: Stroke Impairment Assessment Set; VIF: Variance inflation factor; H: Hemorrhagic; I: Ischemic

Parameters	Estimate	SE	t	p-value	VIF
Age	-0.152	0.167	-0.91	0.3654	1.200
Type of stroke (H/I)	5.532	3.331	1.66	0.1011	1.300
Days from onset*	-0.182	0.053	-2.87	0.0054	1.220
FIM motor at admission	0.141	0.121	1.16	0.2500	4.156
FIM cognitive at admission	0.223	0.253	0.88	0.3813	2.342
SIAS motor total at admission	0.183	0.298	0.61	0.5411	3.121
SIAS trunk at admission*	4.656	1.408	3.31	0.0015	3.262

**Table 4:** Results for FIM motor at discharge obtained by multivariate regression analysis in the late elderly group (aged ≥75 years) Statistically significant findings (p<0.05) are marked with an asterisk (*). FIM: Functional Independence Measure; SE: Standard error; SIAS: Stroke Impairment Assessment Set; VIF: Variance inflation factor

Parameter	Estimate	SE	t	p-value	VIF
Age*	-0.766	0.262	-2.92	0.0045	1.076
Type of stroke (H/I)	0.869	3.097	0.28	0.7796	1.018
Days from onset*	-0.210	0.101	-2.07	0.0420	1.142
FIM motor at admission	0.186	0.157	1.18	0.2399	4.254
FIM cognitive at admission*	0.683	0.223	3.05	0.0031	1.984
SIAS motor total t admission*	0.678	0.338	2.00	0.0486	3.404
SIAS trunk at admission*	4.856	1.527	3.18	0.0021	5.084

## Discussion

In this study, we examined factors associated with functional independence at discharge in patients with stroke, with a particular focus on the prognostic role of age. Using the FIM motor score [13] at discharge as the primary outcome, multivariable regression analyses demonstrated that earlier transfer to a rehabilitation ward and better trunk function were significant predictors of outcome in the early elderly group, whereas these factors, together with age and cognitive function, were independently associated with outcomes in the late elderly group.

Notably, age was a significant predictor in the overall cohort (estimate, −0.354) and showed a stronger association in the late elderly group (estimate, −0.766), but was not significant in the early elderly group. These findings suggest that the influence of age on functional recovery becomes more pronounced in patients aged ≥75 years. The present findings support the clinical relevance of the Japanese distinction between early and late elderly populations when considering stroke rehabilitation prognosis.

One possible explanation for the lack of an age effect in the early elderly group is the greater recovery capacity observed in this population, regardless of baseline impairment severity. In addition, a ceiling effect of the FIM may have limited its sensitivity in detecting higher levels of functional independence among early elderly patients; notably, 20% of early elderly patients achieved the maximum FIM motor score at discharge, whereas none in the late elderly group did. These findings suggest that more sensitive outcome measures may be required in future studies.

Using a cohort from a rural region of Japan, where population aging is particularly advanced, this study demonstrated that the influence of age on post-stroke outcomes differs between patients aged ≥75 years and those aged <75 years. Although individuals aged ≥65 years have been defined as elderly in Japan since 1956, increases in both life expectancy and healthy life expectancy necessitate reconsideration of this definition. The United Nations report World Population Ageing 2019 proposed the prospective old-age dependency ratio, defining old age as the age at which remaining life expectancy is 15 years [15,16]. Importantly, although demographic trends have continued to evolve following the COVID-19 pandemic, these reports remain foundational references for defining population aging. In Japan, this threshold was estimated to be 73.8 years in 2021, closely aligning with the proposal by Orimo et al. to classify individuals aged ≥75 years as elderly [17,18].

The explanatory variables included in the regression analyses were selected based on previous studies identifying age, stroke subtype, motor impairment severity, trunk function, and cognitive function as key predictors of FIM motor outcomes [6,19-21]. Time from stroke onset to transfer was included because early intensive rehabilitation has been shown to positively influence functional recovery [22]. Although recurrent stroke has been reported to affect functional independence at discharge, it was not included as a variable because patients with substantial effects of recurrence were effectively excluded by limiting inclusion to those with low Fazekas scores [11]. Despite the observed difference in sex distribution between groups, it was not included in the regression models because previous studies have reported inconsistent associations between sex and post-stroke functional outcomes after adjustment for baseline characteristics. In addition, exploratory analyses in the present study showed that inclusion of sex did not materially influence model estimates. Therefore, to avoid model overfitting relative to the sample size, sex was excluded from the final regression models [23,24].

In the late elderly group, lower FIM scores at admission were associated with lower FIM scores at discharge. However, this finding should not be interpreted as evidence that rehabilitation is ineffective in older patients. Previous studies have demonstrated meaningful functional improvements in this population [25]. In the present study, the mean FIM gain in the late elderly group was 27 points, exceeding values reported in earlier studies [26]. This relatively large gain may be attributable to the inclusion of only patients who were independent prior to stroke onset [27].

Several limitations should be acknowledged. First, cases of recurrent stroke were limited to those with mild severity, and patients with severe recurrence were excluded. Second, patients with advanced dementia were excluded, resulting in a restricted sample size. Additionally, patients with subarachnoid hemorrhage and those with cerebellar or brainstem lesions were excluded because these conditions often present with distinct clinical features. Future studies incorporating broader stroke subtypes and cognitive profiles are warranted. The relatively modest sample size in relation to the number of predictors may have introduced a risk of model overfitting, and findings should therefore be interpreted cautiously.

## Conclusions

In patients undergoing post-stroke rehabilitation, the influence of age on functional outcomes differs by age group. Age was not a significant predictor of functional independence at discharge among patients aged <75 years, but emerged as a strong and independent determinant among those aged ≥75 years. These findings suggest that chronological age alone should not be overemphasized when predicting outcomes or setting rehabilitation goals in the early elderly. In contrast, among late elderly patients, age-related factors interact with cognitive function, motor impairment, and trunk control, underscoring the need for age-specific prognostic models. Incorporating age-stratified prognostic approaches into rehabilitation planning may improve goal setting, resource allocation, and shared decision-making in super-aging societies such as Japan. These findings should be interpreted within the context of patients undergoing intensive rehabilitation in the convalescent phase and meeting the specific inclusion criteria applied in this study.
